# Domestic Correspondence

**Published:** 1890-02

**Authors:** 


### Domestic Correspondence.

To The Editor:

*Louisiana State Dental Society.*—The Fifth annual meeting of the Louisiana State Dental Society will be held in New Orleans, on February, 19, 20, and 21, 1890. The programme will consist of reports from committees, essays, clinics, and display of dental specialties. Members of the dental profession are invited.

J. G. McCulloch,
*Recording Secretary.*

New Orleans, January 2, 1890.

---

To the Editor:

The Seventh Annual Convention of the Maryland State Dental Association finished its work and adjourned yesterday evening (December 7), after a lantern exhibit and lecture on "Life from a Biological Point of View," by Dr. W. Xavier Sudduth, of Philadelphia. The business sessions in the morning and afternoon were devoted chiefly to the giving of clinics, the reading of papers, and the election of officers, a great deal of time being devoted to the discussion of Dr. Cyrus M. Gingrich's paper on "Operative Dentistry." In Dr. B. Holly Smith's paper, on "Dental Literature," he congratulated the society on the high order of merit which the dental journals have attained. The following officers were elected and installed: Dr. A. B. Scott, president; Dr. A. B. King and Dr. John C. Uhler, vice-presidents; Dr. W. W. Dunbracco and Dr. J. J. Williams, secretaries, Dr. T. H. Davy, treasurer, and Dr. Cyrus M. Gingrich, Dr. A. Price and Dr. A. P. Gore, executive committee. Dr. W. B. Winder, of the Baltimore Dental College, made a short speech, urging members of the association to join the National Dental Protective Association. Dr. A. P. Gore, Dr. A. J. Volk, and Dr. F. F. Drew were appointed to take notice of any violation of the code of ethics and report to the chairman in sealed envelopes. A committee, consisting of Dr. Winder, Dr. Wm. A. Mills, and Dr. Bernard Meyer, was appointed to look into the affairs of the defunct Odontological Society and transfer any funds belonging to it to the Maryland Association. Clinics were given by Dr. S. H. Guilford, of Philadelphia, on "Method of Staining and Coloring of Artificial Teeth;" by Dr. Wm. H. Gingrich, of Norfolk, on "The Treatment of Approximal Surface of Molars and Inserting Compound Contour Gold Fillings;" and by Dr. David Genese, of Baltimore, on "The Attachment of Pivotal Crowns of a New Form."

